# Resident Physicians’ Knowledge and Preparedness Regarding Human Monkeypox: A Cross-Sectional Study from Saudi Arabia

**DOI:** 10.3390/pathogens12070872

**Published:** 2023-06-26

**Authors:** Ali Mohammad Shafei, Khalid M. Al-Mosaa, Najm Z. Alshahrani, Mohammed Hassan Mohammed ALAmmari, Mashael Obaid Othman Almuhlafi, Nasser Hassan Awdah Al Draim, Afnan Misfer Alwadie, Abdullah Ibrahim Alghrab

**Affiliations:** 1 Asir Central Hospital, Ministry of Health, Abha 62461, Saudi Arabia; 2 Consultant of Preventive Medicine, Saudi Board of Preventive Medicine-Aseer Region, Ministry of Health, Abha 62527, Saudi Arabia; 3 Department of Family and Community Medicine, Faculty of Medicine, University of Jeddah, Jeddah 21589, Saudi Arabia; 4 Public Health Department, Wadi Bin Hashbel Sector, Ministry of Health, Khamis Mushait 61961, Saudi Arabia; 5 Public Health Department, Khamis Mushait Health Sector, General Directorate of Health Affairs in Asir Region, Ministry of Health, Khamis Mushait 62458, Saudi Arabia; 6 Vector Borne and Zoonotic Diseases Administration, Public Health Department, General Directorate of Health Affairs in Asir Region, Ministry of Health, Abha 62458, Saudi Arabia; 7 King Fahad Armed Forces Hospitals Southern Region, Khamis Mushait 64262, Saudi Arabia; 8 Riyadh Third Health Cluster, Diriyah Hospital, Ministry of Health, Riyadh 7717, Saudi Arabia

**Keywords:** Mpox, monkeypox, knowledge, confidence, preparedness, physicians, Saudi Arabia

## Abstract

This study aimed to evaluate knowledge about monkeypox and confidence in dealing with monkeypox diagnosis and management among resident physicians in the Asir region of Saudi Arabia. The data of this cross-sectional study were collected online through a structured questionnaire (N = 315). Knowledge about monkeypox was assessed by 24 questions and a three-item scale assessed confidence in managing monkeypox cases. Multiple logistic regression analysis was performed to assess the associations. Overall, two-thirds of the respondents (67.3%) showed good knowledge about monkeypox and the rest of them (32.7%) had poor knowledge. Respondents who received a copy of the Saudi MOH protocol for monkeypox and attended any conference or lecture about monkeypox were more likely to have good knowledge about monkeypox. Moreover, almost two-thirds of the participants were not confident that they could diagnose or manage the monkeypox. Respondents who received information about monkeypox during residency or medical school had higher confidence in managing monkeypox cases. It can be concluded that the Saudi healthcare system has scope to take necessary steps to contain the monkeypox endemic. The Saudi MOH should arrange conferences and educational programs on monkeypox so that healthcare professionals can improve their knowledge and be confident in the management and diagnosis of monkeypox cases.

## 1. Introduction

Monkeypox (Mpox) virus, a Poxviridae family virus, is now regarded as an international concern. Though it primarily causes small outbreaks or endemics, there are concerns that it might cause the next epidemic if people and the medical world remain ill prepared. Mpox is a zoonotic infection, and various rodents and primates in Africa serve as its reservoirs. It is transmitted in two ways: (i) humans to animals or (ii) humans to humans [1,2]. Its human-to-human transmission occurs through close contacts like skin-to-skin, respiratory droplets, and sexual contact. In the current 2022 outbreak, it has particularly affected men who have sex with men [1,2]. However, it would be unfair to consider it merely a problem of a specific population group, and there is a significant risk that it may become a significant outbreak and spread, thus influencing the broader population and even causing significant mortality [1,2,3,4].

Mpox has an incubation period of 5–21 days, a prodromal period of 1 to 4 days, and a rash period of 14–28 days [2,3]. It causes symptoms quite similar to smallpox, albeit less severe, and has a lower mortality rate [1]. It causes symptoms like prodromal fever, fever often between 38.5 °C and 40.5 °C, malaise, headache, lymphadenopathy, and lesions on the palms and soles. Lesions are often hard, deep, well circumscribed, and umbilicated [5]. Mpox is contagious once symptoms begin and remains contagious until lesions disappear. It may also have an atypical presentation in some individuals. Thus, in some, it may even cause neurological symptoms associated with high mortality rates. Its diagnosis is mainly confirmed through positive PCR tests of skin or mucosal lesions swabs [6].

Mpox was first identified in crab-eating macaques in Denmark in 1958 [1]. However, the first human virus outbreaks were identified in Central and West Africa in the 1970s. Since then, many endemics of Mpox have occurred in the region. It appears to affect about 10% of individuals not vaccinated against smallpox. It has an overall fatality rate of 1% to 11% [5,6,7]. However, in recent years it has been spreading to other parts of Africa, causing much concern. Thus, the 2017 and 2018 outbreaks in Nigeria and Cameroon have caused significant concern regarding the changing face of the virus [7]. Moreover, since 2003, due to high rates of international travel, many sporadic cases of Mpox have been reported outside Africa. This has also led to an increase in rates of human-to-human transmission. This only highlights the importance of developing a better understanding of the virus [6,7].

However, the 2022 global outbreak of Mpox has come as a surprise to most experts. Its cases have been widely reported in regions that have never previously experienced Mpox infections. This has led to the World Health Organization (WHO) declaring the Mpox outbreak a public health emergency [8]. There are widespread Mpox cases with upwards of 86,000 cases worldwide [9]. It would be unwise to think that further outbreaks may not happen, especially considering that the virus is changing and the number of people vaccinated for smallpox is declining due to its discontinuation [10]. Despite many mechanisms in place, researchers have not been able to anticipate this emerging threat [11]. Studies suggest that although the COVID-19 pandemic might have improved awareness, various health systems are still unprepared for the next pandemic [12,13]. The current response occurs too late, and this provides viruses sufficient time to evolve in humans after primary infection. Further, delayed response to the outbreaks amplifies the epidemic [14].

Recently, several studies have been conducted on the knowledge and awareness of Mpox infection among different population groups in Saudi Arabia and other parts of the world. There is evidence of low knowledge regarding human Mpox among Bangladeshi medical doctors [15]. A previous study found lower knowledge about Mpox among healthcare workers in Jordan [16]. This study also reported that healthcare workers’ confidence levels were lower for the ability to manage and diagnose human Mpox cases based on their current level of knowledge and skills [16]. Another study was carried out among internal medicine residents in Palembang, Indonesia, and found that they had quite good attitudes and perceptions regarding Mpox, but their knowledge was not very good [17]. Alshahrani et al. [18] conducted a cross-sectional study among undergraduate medical students (n = 324) from King Khalid University, Abha, Saudi Arabia, and found that the vast majority (72%) of them had poor knowledge about human Mpox infection. This is despite the fact that nearly half of the participants agreed that Mpox posed a significant threat to public health [19]. The findings were equally discouraging in another cross-sectional study by Alshahrani et al. [20] among healthcare workers in Saudi Arabia. Out of the 398 participants, just 18.6% of physicians were confident in managing Mpox. Most physicians lacked knowledge regarding endemicity and the transmission of Mpox. In addition, they had insufficient knowledge to differentiate Mpox infection from chickenpox [21]. Therefore, there is little surprise that the general Saudi population also has a poor understanding of the condition. Thus, a cross-sectional study carried out from May to July 2022 assessing the knowledge of the general population in Saudi found that only about half of the participants had some understanding of the condition [22].

Although Saudi Arabia has not experienced a major outbreak like many other nations, and only a few cases (less than 10) have been reported, this does not mean that Mpox does not threaten the nation [23]. There are many reasons why the Saudi healthcare system must be concerned about Mpox. First, numerous large-scale outbreaks have occurred outside Africa for the first time, affecting many people. Second, it has started spreading from human to human. Thirdly, its virulence has changed due to mutations. Fourth, it poses a greater risk to specific population groups. Fifth, it highlights major gaps in understanding viral transmission dynamics [24]. All this means that Saudi Arabia must prepare to counter the Mpox outbreak. Since healthcare workers play a vital role in countering any epidemic, understanding their preparedness for emerging diseases or conditions is crucial. Thus, it is important to explore how prepared Saudi healthcare workers are in preventing and managing the condition. To address the knowledge gap and provide evidence in this regard, the current study, therefore, aimed to evaluate knowledge about human Mpox and confidence in dealing with Mpox diagnosis and management among resident physicians in the Asir region of Saudi Arabia.

## 2. Materials and Methods

### 2.1. Study Design, Settings, and Subjects

This was an analytical cross-sectional study with the primary objective to determining the preparedness for managing human Mpox infection of resident physicians under the Saudi Commission for Health Specialties (SCFHS) in the Asir region of Saudi Arabia during the academic year of 2023. In this study, “preparedness” was defined as the comprehensive knowledge, skills, and abilities required to provide care and prevent disease.

This study was conducted in various governmental healthcare facilities in the Asir multi-center regions of Saudi Arabia, including Khamis Mushait General Hospital, Khamis Mushait Maternity and Children Hospital, Armed Forces Hospitals in the Southern Region, Asir Central Hospital (ACH), Abha Maternity and Children Hospital, and King Khalid University Medical City.

### 2.2. Sample Size, Sampling, and Data Collection Techniques

The target population consisted of 1200 physicians undergoing postgraduate training in the Asir region under the SCFHS. The minimum sample size required for this study was calculated by the single-sample proportion test. The following assumptions were considered: (i) 55% of physicians had good knowledge about Mpox based on a previous study in Saudi Arabia [20], (ii) a confidence level of 95%, and (ii) a margin of error of 5%. Thus, a sample size of 289 resident physicians was estimated.

A simple random sampling procedure was applied to enroll the participants. We collected a list of 1200 trainees from the SCFHS, which contained contact details such as email addresses. All individuals’ information was inserted into an Excel spreadsheet (i.e., 1200 individuals comprised the sampling frame) and a random sample was generated from the sampling frame using Excel. Thus, 500 resident physicians were randomly selected for sending invitations to participate in the study. Finally, 315 resident physicians consented to participate in this study (i.e., sample size of this study was 315).

An online technique was used to gather the survey data. Three research staff distributed invitation links to the randomly selected resident physicians (n = 500) to complete the survey via social media platforms (such as Telegram, WhatsApp, etc.) and email. The questionnaire was attached to the invitation link in the form of a Google survey file. The survey questionnaire was disseminated between 1 March and 1 May 2023. The questionnaire was designed in English and all survey items were required to be answered to minimize non-response bias. Approximately, 8–12 min was required to complete each response. Data were stored in Excel for subsequent statistical analysis.

### 2.3. Survey Contents

A structured questionnaire was used in this study, which was based on the previous literature [18,20,21,22]. Some parts of the questionnaire were previously used in Saudi Arabia (e.g., knowledge about Mpox) [18,20]. The survey questionnaire consisted of three sub-sections: (i) sociodemographic and profession-related information, (ii) knowledge about human Mpox, and (iii) confidence in dealing with monkeypox diagnosis and management (see Supplementary File S1). Sociodemographic and profession-related information, such as age, gender, marital status, level of work, work center, nature of work, etc., were included. Moreover, knowledge about human Mpox was assessed by 24 questions which included knowledge about the epidemiology, clinical aspects, transmission, and treatment of the disease [18,20,21,22]. Knowledge score was computed by summing up the 24 knowledge items with correct responses assigned as “one” point and incorrect responses assigned as “zero”. The mean knowledge score was set as a cutoff value for categorizing the overall knowledge. An individual who scored above the mean knowledge score was categorized as having “good knowledge”. Finally, confidence in human monkeypox diagnosis and management was assessed by three items, retrieved from Harapan et al. [25]. The two available answers were “yes” or “no,” with “yes” receiving a score of one and “no” receiving a score of zero. A total score is a range of 0 to 3, with higher scores signifying greater confidence.

### 2.4. Pilot Study and Reliability of the Questionnaire

Before administrating the final version of the questionnaire, a pilot study was conducted among fifteen residents undergoing training in different specialties, and necessary modifications were made to the questionnaire based on feedback from a committee of twelve medical consultants/specialists. Hence, the questionnaire was easily understandable and clear for the population group. Moreover, reliability analysis was performed using Cronbach’s alpha to assess the internal consistency of the study variables. The Cronbach’s alpha for the knowledge section was 0.929 and for the confidence was 0.834. A good level of internal consistency was found for both sections, indicating that the questions within each section are highly related and measure the same construct.

### 2.5. Data Analysis

Data were analyzed by statistical package for the social sciences (SPSS) version 28. Descriptive statistics (such as frequency, percentage, mean, etc.) were calculated to summarize the data. The chi-square/Fisher’s exact test was used to examine associations between dependent variables. Finally, logistic regression analysis was performed to determine predictors of good knowledge and high confidence. *p* values of <0.05 were considered statistically significant.

### 2.6. Ethics

Ethical approval was obtained from the Abha Health Ethics Committee and the Saudi Commission for Health Specialties, Asir Region (IRB Log No: REC-4-2-2023). Informed consent was taken from all participants before their study enrollment. Participation was self-nominated and did not offer any incentives. Participants were assured that participation was anonymous. Any identifying information, such as participants’ names, contact numbers, etc., was not asked for or not disclosed publicly. The confidentiality of their personal information was strictly maintained, and it was ensured that the data were used only for research purposes.

## 3. Results

### 3.1. Sociodemographic and Profession-Related Information

This study comprised 315 samples, of which two-thirds (66%) were male. The majority of the respondents (80%) were between 25 and 34 years of age. Approximately a quarter of the respondents (27.9%) had more than five years of medical practice experience. Only 36.2% of respondents received an Mpox infection management protocol copy from the Saudi MOH. Respondents’ sociodemographic and profession-related information is depicted in Table 1.

### 3.2. Source of Information

**Figure 1:**
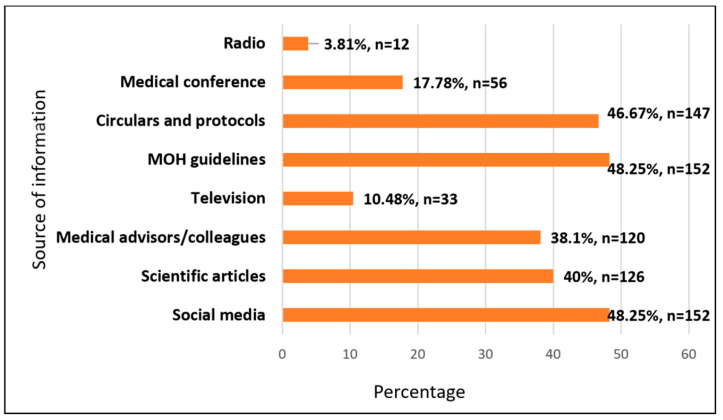
Sources of information about Mpox among study participants (N = 315).

Almost half of the respondents (48.25%) reported that their primary source of information about monkeypox was MOH guidelines and social media. Other relevant sources were circular and protocols (46.67%), articles (40%), colleagues (38.10%), medical conferences (17.78), television (10.48%), and radio (3.81%) (Figure 1).

### 3.3. Knowledge about Mpox and Its Associated Factors

The assessment of knowledge regarding human Mpox is summarized in Table 2. Approximately half of the respondents knew about the high prevalence of Mpox in Western and Central Africa, and more than half did not have much knowledge about its transmissibility. Similarly, half of the participants had some understanding of its symptoms and disease presentation. The study also found significant gaps in the knowledge regarding disease prevention and management (Table 2). Overall, two-thirds of the respondents (67.3%) showed a good level of knowledge about monkeypox and the rest of them (32.7%) had poor knowledge. The mean knowledge score was 14.0 (SD: 6.4).

Table 3 shows the association between demographic variables and knowledge about Mpox. Bivariate analysis found that participants’ age (*p* = 0.01) and level of work (*p* < 0.01), and identifying as dentists were indicative of significantly poorer knowledge compared to those who identified as preventive medicine specialists (*p* = 0.001), who engaged in routine work (*p* = 0.01), had medical practice experience (*p* = 0.01), received information about Mpox during residency or medical school education (*p* = 0.02), had a copy of the Saudi MOH Protocol (*p* < 0.01), had received information earlier (*p* < 0.01), and attended a conference (*p* = 0.01) were significantly associated with having a good knowledge score for Mpox.

The multiple logistic regression model revealed that respondents who received a copy of the Saudi MOH protocol for patients suspected/confirmed with monkeypox had a good level of knowledge about monkeypox compared to their counterparts (Adjusted Odds Ratio (AOR) = 4.25, 95% Confidence Interval (C.I.) = 2.49 to 5.64). Moreover, respondents who attended any conference or lecture about Mpox were more likely to have good knowledge about Mpox compared to their counterparts (AOR = 3.34, 95% C.I. = 2.12, 6.96) (Table 3).

### 3.4. Confidence in the Management and Diagnosis of Monkeypox

Regarding the confidence in diagnosing and managing the condition based on present knowledge, almost two-thirds (61.6% to 72.4%) were not confident that they could diagnose or manage the Mpox (Table 4). Specifically, for the first question, only 27.6% of respondents said they were confident about managing Mpox cases based on their current knowledge and skills, while the majority (72.4%) said they were not confident. This suggests a lack of preparedness among respondents when it comes to managing Mpox cases. For the second question, 38.4% of respondents said they were confident about diagnosing Mpox cases based on their current knowledge and skills, while 61.6% said they were not confident. This indicates that a significant number of respondents may not have the necessary knowledge and skills to diagnose Mpox cases accurately. Finally, for the third question, only 35.6% of respondents said they were confident about diagnosing Mpox cases based on the ability of their current facility to perform diagnostic tests, while 64.4% said they were not confident. This suggests there may be issues with the availability or reliability of diagnostic tests for Mpox in the respondents’ facilities.

Multiple logistic regression analysis found a significant association between the level of work and confidence with dealing with Mpox patients, as those in senior levels, i.e., R4, had the highest confidence compared to all other junior levels of work, i.e., R1 and R2 (AOR = 5.15, 95% CI = 1.19 to 13.68), with (*p* = 0.001). Regarding medical specialty, dermatology residents had a higher confidence in dealing with Mpox compared to their counterparts (AOR = 1.70, 95% CI = 1.06 to 2.73). Moreover, respondents who received information about Mpox during residency or medical school (AOR = 2.26, 95% CI = 1.18 to 3.89) and working in administrative roles (such as General Directorate, Weqaa, Gulf CDC, etc.) (AOR = 2.27, 95% CI = 1.16 to 4.45) had a higher confidence compared to their counterparts (Table 5).

### 3.5. Medical Specialties

**Figure 2:**
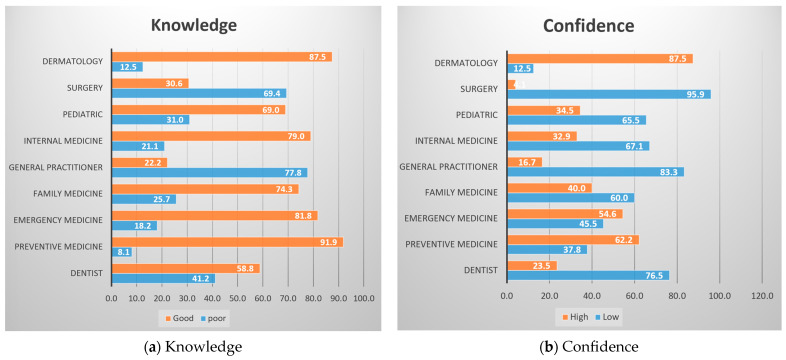
(**a**) Displays the association between medical specialties of the participants and their level of knowledge. (**b**) Displays the association between medical specialties of the participants and their level of confidence.

The relationship between study participants’ medical specialties and their level of knowledge about Mpox is shown in Figure 2a. The figure shows that participants with medical specialties in preventive medicine (91.9%), dermatology (87.5%), emergency medicine (81.8%), internal medicine (79%), and family medicine (74.3%) were more likely to report having a good understanding of monkeypox. Participants with medical specialties in other areas, such as general practice (22.2%) and surgery (30.6%), were less likely to report having a good knowledge about Mpox.

Figure 2b shows the confidence in dealing with Mpox according to medical specialties; it shows that medical specialties may play a role in the level of confidence that healthcare providers have in their ability to diagnose and manage Mpox cases. Healthcare providers with medical specialties in dermatology (87.5%), preventive medicine (62.2%), emergency medicine (54.6%), and family medicine (40%) may be more likely to have received training and experience in the diagnosis and management of Mpox cases. As a result, they may be more confident in their ability to provide care for patients with Mpox.

## 4. Discussion

To control any epidemic in the early stages, the knowledge and preparedness of healthcare organizations play a crucial role. Healthcare workers must be prepared to identify any outbreak in its early stages, initiate preventive measures, and isolate and manage those affected by the infection. However, lapses in this early control mechanism may result in the spread of infection and thus result in an epidemic. Identifying the gaps in the knowledge and preparedness of healthcare workers regarding Mpox infection is the first step in preventing this endemic disease from becoming a pandemic. Therefore, our study evaluated knowledge about human Mpox and confidence in managing Mpox cases among resident physicians in the Asir region of Saudi Arabia.

In our sample, two-thirds of the resident physicians (67.3%) had a good level of knowledge about human Mpox. The overall prevalence of good knowledge is higher than that of reported in a previous study conducted among general practitioners in Indonesia [21]. They reported that around one-third (36.5%) of the study participants had good knowledge about Mpox (based on a 70% cutoff point for the knowledge domain) [21]. Another study reported that clinicians in Ohio have poor levels of knowledge about Mpox [26]. A recent study by Alshahrani and colleagues, carried out among physicians in Saudi Arabia, revealed that more than half (55%) of the study subjects had a good level of knowledge about human Mpox [20]. However, our study found a significant number (32.7%) of resident physicians had poor knowledge about Mpox, which represents a matter of concern for the country’s healthcare systems. For instance, our study found more than half of the participants had less knowledge about monkeypox’s transmissibility. It is imperative that physicians have better knowledge of Mpox so that they can develop the capacity to treat human Mpox cases.

Based on the findings from the regression model, resident physicians receiving a copy of the Saudi MOH protocol for patients suspected/confirmed with Mpox and attending any conference or lecture about Mpox were predictors of good knowledge about Mpox. These findings suggest that arranging conferences and lecture programs on the preparedness for human Mpox would be effective in improving knowledge about Mpox.

Another key finding of this study is that almost two-thirds of the study physicians were not confident that they could diagnose or manage Mpox. Evidence shows that Saudi physicians had poor attitudes towards Mpox [20]. A recent study from Indonesia reported that general practitioners had a low level of confidence to diagnose and manage Mpox cases based on their current knowledge, skills, and facilities [25]. The previous literature reported that knowledge and attitudes regarding Mpox are positively correlated, indicating that good knowledge showed a significant association with positive attitude [15]. Future explanatory studies are recommended to observe whether there is a relationship between knowledge, attitudes, and confidence levels.

Our study also found that respondents who received information about Mpox during residency or medical school and worked in administrative roles (such as General Directorate, Weqaa, Gulf CDC, etc.) had a higher confidence in managing Mpox cases. Another study also found that general practitioners are likely to be more confident if they received education about Mpox during their education in medical school [16,25]. Since informal information may produce uneven results, learning from a formal curriculum during medical education would offer a better learning experience [27,28].

An important finding is that dermatology residents have a higher confidence in dealing with Mpox. Because of the predominant skin symptoms of Mpox (including anogenital skin lesions), dermatologists can play vital role in identifying new Mpox cases, assisting in the diagnosis and prevention of disease spread through identification and isolation, contact tracing, and education [29,30]. In the case of knowledge about Mpox, participants with medical specialisms in preventive medicine and dermatology have also showed a higher prevalence of good knowledge about Mpox. However, in the regression model, there was no significant association between knowledge about Mpox and dermatology residents. A small sample size of dermatology respondents may have influenced the results, leading to a higher risk of skewing. Further studies, which may include a representative sample of all medical specialists, are recommended to better understand the knowledge gap regarding human Mpox among medical specialists. Moreover, the adjusted regression model showed that respondents who worked at a senior level (R4) had higher confidence compared to those who worked at the R1 level (junior level). These findings imply that experienced health professionals may have greater knowledge and confidence about a certain disease. A previous Turkish study reported that older physicians had greater knowledge about Mpox [31]. However, in our study, age was not statistically significant in terms of knowledge and confidence about Mpox.

Further, the study regarding the preparedness of the Saudi Healthcare sector for countering Mpox is justified based on the reports from other regions and also early studies covering Saudi healthcare. For example, reports show that one of the reasons for the significant spread of the infection in Latin America has been an insufficient response to infection. Despite the availability of some experimental drugs like cidofovir and the known effectiveness of the classical smallpox vaccine in countering the spread, none have been available in the region and the medical fraternity has mainly been unprepared [24]. Similar causes of outbreaks in Portugal, the UK, and Nigeria have been identified, that is, ill preparedness and the poor knowledge of medical staff in the nations resulting in poor and delayed responses to the outbreak [32,33]. Similarly, findings from a cross-sectional study of general practitioners in Indonesia are also discouraging. In the study, 88.8% of physicians agreed that there is a need to understand the condition better, as most physicians lack confidence in diagnosing and treating the condition based on their current skills [21].

### 4.1. Implications of the Findings

Since this study was one of the first studies on the topic in Saudi Arabia, the findings provide baseline evidence and contribute to the literature. Policymakers can use the findings to develop potential steps for improving the preparedness against Mpox as an initial preventive measure. An effective way to prepare for an Mpox outbreak in Saudi Arabia is to develop and implement practical workshops and hands-on courses about emerging Mpox to improve the knowledge and skills of medical professionals. In addition, the Saudi MOH should disseminate updated information regarding Mpox among health professionals as well as the general public through newspapers, websites, and social media platforms.

### 4.2. Limitations of This Study

The use of a cross-sectional design limits the ability to infer causality from the results. The reliance on self-reported data may introduce bias, as participants may overestimate or underestimate their knowledge and preparedness. Future studies could consider using objective measures, such as knowledge tests or assessments of actual performance in simulated scenarios. The sample may not be representative of all resident physicians in Saudi Arabia, limiting the generalizability of the findings.

## 5. Conclusions

Our findings conclude that the Saudi healthcare system has scope to take necessary steps to contain the Mpox endemic. Approximately one-third of the resident physicians had poor knowledge about monkeypox, and two-thirds of them had a lack of confidence and skills to diagnose and manage the condition on time, which is critical in preventing the spread of infection. These findings highlighted that the Saudi MOH should arrange conferences or lecture programs, educational programs, and information dissemination on monkeypox so that healthcare professionals can improve their knowledge and be confident in the management and diagnosis of Mpox cases.

## Figures and Tables

**Table 1:** Sociodemographic-related information of study participants (N = 315).

Variable(s)	Frequency	Percentage
Age (in years)		
25–29	141	44.8
30–34	135	42.9
35–39	34	10.8
40 and more	5	1.6
Gender		
Male	208	66.0
Female	107	34.0
Marital status		
Single	138	43.8
Married	177	56.2
Level of work		
R1	59	18.7
R2	51	16.2
R3	73	23.2
R4	98	31.1
R5	12	3.8
GP	22	7.0
Medical Specialty		
Dentist	17	5.4
Preventive Medicine	37	11.8
Emergency Medicine	11	3.5
Family medicine	70	22.2
General practitioner	18	5.7
Internal Medicine	76	24.1
Pediatric	29	9.2
Surgery	49	15.6
Dermatology	8	2.5
Where is your work center?		
University KKU-Hospital	14	4.4
Khamis Mushait Health Sector “MOH”	63	20.0
Military Health Sector “AFHSR or MOI	76	24.1
Abha Health Sector “MOH”	162	51.4
Nature of your routine work in hospital/health center/clinic?		
Outpatient	65	20.6
Inpatient and outpatient	155	49.2
Others	37	11.7
Inpatient	24	7.6
Administrative *	34	10.8
Medical practice experience in years		
<1 year	34	10.8
1–5 years	193	61.3
>5 years	88	27.9
Had you ever received information about Mpox during residency or medical school education?		
Yes	124	39.4
No	191	60.6
Did you receive a copy of the Saudi MOH Protocol for Patients Suspected/Confirmed with Mpox?		
Yes	114	36.2
No	201	63.8
When was the first time you heard information about Mpox?		
Within last several days or weeks	4	1.3
Within last month or later	36	11.4
Within last 6 months or later	260	82.5
I did not hear about it	15	4.8
Did you attend any conference or lecture about Mpox?		
Yes	73	23.2
No	242	76.8

* Those who worked in General Directorate of MOH, Weqaa, and Saudi/Gulf CDC offices.

**Table 2:** Items for assessment of knowledge regarding human Mpox.

Items	Response, N (%)	
	Yes	No
1. Mpox is prevalent in middle eastern countries	56 (17.78)	**259 (82.22)**
2. Mpox is prevalent in Western and Central Africa	**148 (46.98)**	167 (53.01)
3. There are human Mpox cases in Saudi Arabia	**143 (45.39)**	172 (54.6)
4. Is Mpox a viral disease infection?	**286 (90.79)**	29 (9.2)
5. Mpox is easily transmitted via droplet contact (e.g., sneezing, coughing, etc.)	**172 (54.60)**	143 (45.39)
6. Mpox could be transmitted through intimate contact	**212 (67.30)**	103 (32.69)
7. Is Mpox easily transmitted human-to-human?	**189 (60)**	126 (40)
8. Could Mpox be transmitted through a bite of an infected monkey?	142 (45.07)	**173 (54.92)**
9. Travelers from America and Europe are the main source of imported cases of Mpox	126 (40)	**189 (60)**
10. Mpox and smallpox have similar signs and symptoms	**148 (46.98)**	167 (53.01)
11. Mpox and chickenpox have similar signs and symptoms?	136 (43.17)	**179 (56.8)**
12. A Flu-like syndrome is one of the early signs or symptoms of human Mpox?	**206 (65.39)**	109 (34.6)
13. Rashes on the skin are one of the signs or symptoms of human Mpox?	**238 (75.56)**	77 (24.44)
14. Mpox is diagnosed by taking a swab sample from the lesion for a polymerase chain reaction	**191 (60.63)**	124 (39.36)
15. A person does not exhibit symptoms during the incubation period	115 (36.50)	**200 (63.49)**
16. Incubation period of Mpox lasts between 3–5 days	101 (32.21)	**214 (67.93)**
17. Diarrhea is one of the signs or symptoms of human Mpox?	88 (27.93)	**227 (72.06)**
18. Lymphadenopathy (swollen lymph nodes) is one clinical sign or symptom that could be used to differentiate Mpox and smallpox cases?	**179 (56.82)**	136 (43.17)
19. There is a specific treatment for Mpox?	67 (21.26)	**248 (78.73)**
20. Analgesics and antipyretics can be given to manage local pains and fever caused by Mpox	**236 (74.92)**	79 (25.07)
21. Antivirals are required in the management of human Mpox patients	**151 (47.93)**	164 (52.06)
22. Antibiotics are required in the management of human Mpox patients	28 (8.89)	**287 (91.11)**
23. People who got chickenpox vaccine are immunized against Mpox	102 (32.38)	**213 (67.61)**
24. There is a specific vaccine for Mpox?	**54 (17.14)**	261 (82.85)

Note: Bolded values represent the correct answer.

**Table 3:** Factors associated with good level of knowledge regarding Mpox among study participants.

Variables	Categories	Knowledge		Chi-Square Test/Fisher’s Exact Test	Regression Model			
Overall knowledge score		Poor	Good	*p*	AOR	95% C.I.		*p*
		103 (32.7)	212 (67.3)					
Age groups	25–29	59 (41.8)	82 (58.2)	0.01 *	Ref.			
	30–34	37 (27.4)	98 (72.6)		0.59	0.04	11.36	0.837
	35–39	6 (17.6)	28 (82.4)		1.05	0.08	14.34	0.985
	40 and more	1 (20.0)	4 (80.0)		1.33	0.09	17.58	0.912
Gender	Female	69 (33.2)	139 (66.8)	0.80	Ref.			
	Male	34 (31.8)	73 (68.2)		0.89	0.43	1.86	0.756
Marital status	Married	52 (37.7)	86 (62.3)	0.12	Ref.			
	Single	51 (28.8)	126 (71.2)		1.19	0.57	2.48	0.651
Level of work	R1	30 (50.8)	29 (49.2)	0.01 *	Ref			
	R2	19 (37.3)	32 (62.7)		1.58	0.09	26.94	0.753
	R3	16 (21.9)	57 (78.1)		2.17	0.11	13.91	0.613
	R4	15 (15.3)	83 (84.7)		4.38	0.21	10.37	0.338
	R5	7 (58.3)	5 (41.7)		7.23	0.34	13.61	0.205
	GP	16 (72.7)	6 (27.3)		4.92	0.18	18.29	0.349
Medical Specialty	Dentist	7 (41.1)	10 (58.8)	0.01 *	Ref.			
	Preventive Medicine	3 (8.1)	34 (91.9)		0.624	0.488	0.797	0.001 *
	Emergency Medicine	2 (18.2)	9 (81.8)		1.134	0.900	1.429	0.286
	Family medicine	18 (25.8)	52 (74.3)		0.902	0.568	1.433	0.661
	General practitioner	14 (77.8)	4 (22.2)		0.934	0.672	1.297	0.682
	Internal Medicine	16 (21.1)	60 (78.9)		1.082	0.812	1.442	0.589
	Pediatric	9 (31)	20 (69)		0.912	0.537	1.551	0.735
	Surgery	34 (69.4)	15 (30.6)		0.977	0.626	1.525	0.919
	Dermatology	1 (12.5)	7 (87.5)		1.048	0.880	1.247	0.601
Where is your work center?	University Health Sector	3 (21.4)	11 (78.6)	0.83	Ref.	1		
	Khamis Mushait Health Sector	21 (33.3)	42 (66.7)		1.05	0.14	8.08	0.966
	Military Health Sector	26 (34.2)	50 (65.8)		1.24	0.45	3.41	0.672
	Abha Health Sector	53 (32.7)	109 (67.2)		1.07	0.49	2.35	0.866
Nature of your routine work in hospital/health center/clinic?	Outpatient	16 (24.6)	49 (75.4)	0.01 *	Ref.	1		
	Inpatient and outpatient	65 (41.9)	90 (58.1)		0.71	0.16	3.13	0.648
	Others	11 (29.7)	26 (70.2)		0.52	0.13	2.02	0.341
	Inpatient	7 (29.2)	17 (70.8)		0.26	0.05	1.40	0.118
	Administrative **	4 (11.8)	30 (88.2)		0.70	0.12	4.28	0.700
Medical practice experience	<1 year	18 (52.9)	16 (47.1)	0.01 *	Ref.	1		
	1–5 years	66 (34.2)	127 (65.8)		1.19	0.28	5.09	0.808
	>5 years	19 (21.6)	69 (78.4)		0.87	0.35	2.15	0.758
Received information about Mpox during residency or medical school education	Yes	31 (25.0)	93 (75.0)	0.02 *	Ref.	1		
	No	72 (37.7)	119 (62.3)		0.96	0.44	2.09	0.927
Received a copy of the Saudi MOH Protocol for Mpox management	Yes	13 (11.4)	101 (88.6)	0.01 *	4.25	2.49	5.64	0.001 *
	No	90 (44.8)	111 (55.2)		Ref.	1		
First time hearing information about Mpox	I did not hear about it	1 (33.3)	3 (63.7)	0.01 *	Ref.	1		
	Within last several days or weeks	17 (47.2)	19 (52.8)		0.12	0.09	3.77	0.999
	Within last month or later	74 (28.5)	186 (71.5)		1.91	0.32	11.58	0.480
	Within last 6 months or later	12 (80.0)	3 (20.0)		3.21	0.65	15.81	0.152
Attended any conferences or lectures about Mpox	Yes	15 (20.5)	58 (79.5)	0.01 *	3.34	2.12	6.96	0.001 *
	No	88 (36.4)	154 (63.6)		Ref.	1		

* Statistically significant (i.e., *p* < 0.05). Ref. = reference category. *p* = *p*-value. AOR = Adjusted Odds Ratio. C.I. = Confidence Interval. ** Those who worked in General Directorate of MOH, Weqaa, and Saudi/Gulf CDC offices.

**Table 4:** Confidence in management and diagnosis of Mpox among study participants.

Items	Answer	N	%
1. Are you confident to manage Mpox cases, if any, based on your current knowledge and skills?	Yes	87	27.6
	No	228	72.4
2. Are you confident to diagnose Mpox cases based on your current knowledge and skills?	Yes	121	38.4
	No	194	61.6
3. Are you confident to diagnose Mpox cases based on the ability of your current facility to do diagnostic test?	Yes	112	35.6
	No	203	64.4

**Table 5:** Factors associated with high confidence level in managing and diagnosing Mpox and its predictors among study participants.

Variables	Categories	Confidence		Chi-Square Test/Fisher’s Exact Test	Regression Model			
Overall Confidence score		Low	High	*p*	AOR	95% C.I.		*p*
		206 (65.4)	109 (34.6)			Lower	Upper	
Age groups	25–29	102 (72.34)	39 (27.65)	0.018 *	Ref.	1		
	30–34	86 (63.70)	49 (36.29)		1.44	0.13	16.40	0.768
	35–39	15 (44.11)	19 (55.88)		1.54	0.14	16.42	0.720
	40 and more	3 (60)	2 (40)		2.05	0.18	22.98	0.559
Gender	Female	76 (71.02)	31(28.97)	0.132	Ref.	1		
	Male	130 (62.5)	78 (37.5)		1.41	0.68	2.92	0.362
Marital status	Married	114 (64.40)	63 (35.59)	0.676	Ref.	1		
	Single	92 (66.66)	46 (33.33)		1.50	0.75	3.01	0.251
Level of work	R1	46 (77.96)	13 (22.04)	<0.001 *	Ref.	1		
	R2	37 (72.54)	14 (27.45)		1.33	0.55	3.26	0.528
	R3	47 (64.38)	26 (35.61)		0.57	0.24	1.33	0.192
	R4	46 (46.93)	52 (53.06)		5.15	1.94	13.68	0.001 *
	R5	11 (91.66)	1 (8.33)		1.05	0.79	1.38	0.748
	GP	19 (86.36)	3 (13.63)		1.01	0.54	1.89	0.979
Medical Specialty	Dentist	13 (76.47)	4 (23.52)	<0.001 *	Ref.	1		
	Preventive Medicine	14 (37.83)	23 (62.16)		1.02	0.60	1.75	0.939
	Emergency Medicine	5 (45.45)	6 (54.54)		0.15	0.017	1.34	0.090
	Family medicine	42 (60)	28 (40)		0.82	0.451	1.47	0.499
	General practitioner	15 (83.33)	3 (16.66)		0.99	0.76	1.29	0.924
	Internal Medicine	51 (67.10)	25 (32.89)		0.99	0.88	1.11	0.854
	Pediatric	19 (65.51)	10 (34.48)		0.76	0.47	1.21	0.245
	Surgery	47 (95.91)	2 (4.08)		1.42	0.62	3.23	0.404
	Dermatology	1 (12.5)	7 (87.5)		1.70	1.06	2.73	0.029 *
Where is your work center?	University Health Sector	6 (42.85)	8 (57.14)	0.112 *	Ref.	1		
	Khamis Mushait Health Sector	44 (69.84)	19 (30.15)		3.543	0.63	20.01	0.152
	Military Health Sector	55 (72.36)	21 (27.63)		0.621	0.25	1.56	0.310
	Abha Health Sector	101 (62.34)	61 (37.65)		0.605	0.26	1.41	0.246
Nature of your routine work in hospital/health center/clinic?	Outpatient	40 (61.53)	25 (38.46)	0.005 *	Ref.	1		
	Inpatient and outpatient	116 (74.83)	39 (25.16)		1.19	0.41	3.51	0.744
	Others	22 (59.45)	15 (40.54)		1.34	0.45	4.01	0.602
	Inpatient	11 (45.83)	13 (54.16)		0.96	0.29	3.17	0.947
	Administrative **	17 (50)	17 (50)		2.26	1.18	3.89	0.031 *
Medical practice experience	<1 year	29 (85.29)	5 (14.70)	<0.001 *	Ref.	1		
	1–5 years	131 (67.87)	62 (32.12)		0.31	0.06	1.48	0.141
	>5 years	46 (52.27)	42 (47.72)		0.57	0.26	1.26	0.163
Received information about Mpox during residency or medical school education	Yes	61 (49.19)	63 (50.80)		Ref.	1		
	No	145 (75.91)	46 (24.08)	<0.001 *	2.27	1.16	4.45	0.016 *
Received a copy of the Saudi MOH Protocol for Mpox management	Yes	54 (47.36)	60 (52.63)	<0.001 *	Ref.	1		
	No	152 (75.62)	49 (24.37)		1.48	0.75	2.91	0.261
First time hearing information about monkeypox	I did not hear about it	13 (86.66)	2 (13.33)	0.204	Ref.	1		
	Within last several days or weeks	2 (50)	2 (50)		1.09	0.04	9.93	0.957
	Within last month or later	26 (72.22)	10 (27.77)		1.43	0.18	7.49	0.737
	Within last 6 months or later	165 (63.46)	95 (36.53)		1.58	0.24	5.31	0.634
Attended any conferences or lectures about monkeypox	Yes	29 (39.72)	44 (60.27)	<0.001 *	Ref.	1		
	No	177 (73.14)	65 (26.85)		1.46	0.67	3.18	0.336

* Statistically significant (i.e., *p* < 0.05). Ref. = reference category. *p* = *p*-value. AOR = Adjusted Odds Ratio. C.I. = Confidence Interval. ** Those who worked in General Directorate of MOH, Weqaa, and Saudi/Gulf CDC offices.

## Data Availability

The data presented in this study are available on reasonable request from the corresponding author.

## Supplementary Materials

The following supporting information can be downloaded at https://www.mdpi.com/article/10.3390/pathogens12070872/s1, File S1: Survey: Resident Physicians’ Knowledge and Preparedness Regarding Human Monkeypox: A Cross-Sectional Study from Saudi Arabia.
