# Supplementary material for: Resident Physicians’ Knowledge and Preparedness Regarding Human Monkeypox: A Cross-Sectional Study from Saudi Arabia
Source: Pathogens. 2023 Jun 26;12(7):872. doi: 10.3390/pathogens12070872 (PMC10385241; doi:10.3390/pathogens12070872)
Supplement: Supplementary file 1 [file pathogens-12-00872-s001.zip › pathogens-2453484-supplementary.pdf]

Supplementary File S1: Survey Questionnaire

Title: Resident Physicians' Knowledge and Preparedness Regarding Human Monkeypox: A Cross-sectional Study from Saudi Arabia

Survey Questionnaire

Consent question: -

This survey aims to assess the knowledge about Monkeypox among Saudi physicians. You are then kindly invited to answer the following online survey (on your computer or your mobile phone). We would like to kindly ask you to do your best to answer all questions, if possible, because this will allow us to anonymously process and analyze the data, and thus, improve the knowledge in the field. The survey took about 8 to 12 minutes to complete.

- By ticking in the following case, you acknowledge the following:

• I am Saudi;
• I am a Physician;
• I have read the content of this document including the purpose of the survey/questionnaire;
• I agree to answer, as accurately as possible, the questions of the Survey/ Questionnaire;
• I freely consent to participate to this study.

Do you fully consent to participate in this research? Yes

Socio-demographics questions:

1. Age in years:
2. Gender
   - ✓ Male
   - ✓ Female.
3. Marital status
   - ✓ Married
   - ✓ Single

Workplace questions:

4. Level of work:
   - ✓ Not in SCFHS residency program "service" or GP
   - ✓ Resident R1
   - ✓ Resident R2
   - ✓ Resident R3
   - ✓ Resident R4
   - ✓ Resident R5
   - ✓ Resident R6
5. Medical Specialty

....

6. Where is your work center?
   - ✓ Abha Health Sector "MoH"

✓ Khamis Mushait Health Sector "MoH"
✓ Other Health Sector "MoH" in Aseer region "Mahail, Ahd Rofidah...etc"
✓Military Health Sector "AFHSR or MOI"
✓ University Health Sector "KKU-Hospital or clinic"
✓ Other

7. Nature of **your routine work** in hospital/health center/clinic?
   ✓ Inpatient
   ✓ Outpatient
   ✓ Inpatient and outpatient
   ✓ Administrative "General directorate, Weqaa, Gulf CDC...ect"
   ✓ Others

8. What is/are the main sources of information regarding monkeypox?
   ✓Social media.
   ✓Television.
   ✓Radio.
   ✓ Medical Advisors/ Colleagues.
   ✓Scientific Articles.
   ✓Medical Conference.
   ✓MOH guidelines, circulars and protocols.

Skill of professionals' questions:

9. Medical practice experience:
   ✓ less than 1 year,
   ✓ 1-5 years,
   ✓ More than 5 years.

10. Had you ever received information of human Monkeypox during medical education:
    ✓ Yes,
    ✓No

11. Did you receive a copy of the Saudi MoH Protocol for Patients Suspected/Confirmed with Monkeypox?
    ✓ Yes
    ✓ No

12. When your first time you heard information about Monkeypox:
    ✓ Within several days or weeks ago,
    ✓ Within last month or later,
    ✓ Within last 6 month or later
    ✓ I did not hear about it.

13. Did you attend any conference or lecture about Monkeypox?
    ✓ Yes,
    ✓ No.

Knowledge about Monkeypox questions:

1. Monkeypox is prevalent in middle eastern countries:
   - ✓ Yes,
   - ✓ No.
   - ✓ I don't know.

2. Monkeypox is prevalent in Western and Central Africa:
   - ✓ Yes,
   - ✓ No.
   - ✓ I don't know.

3. There are many human monkeypox cases in Saudi Arabia:
   - ✓ Yes,
   - ✓ No.
   - ✓ I don't know.

4. Is monkeypox a viral disease infection?
   - ✓ Yes,
   - ✓ No.
   - ✓ I don't know.

5. Monkeypox is easily transmitted via droplet contact (eg. sneezing, coughing, etc.)
   - ✓ Yes,
   - ✓ No.
   - ✓ I don't know.

6. Monkeypox could be transmitted through intimate contact

   - ✓ Yes,
   - ✓ No.
   - ✓ I don't know.

7. Is monkeypox easily transmitted human-to-human?
   - ✓ Yes,
   - ✓ No.
   - ✓ I don't know.

8. Monkeypox could be transmitted through a bite of an infected monkey:
   - ✓ Yes,
   - ✓ No.
   - ✓ I don't know.

9. Travelers from America and Europe are the main source of imported cases of monkeypox:
   - ✓ Yes,
   - ✓ No.
   - ✓ I don't know.

10. Monkeypox and smallpox have similar signs and symptoms.
    - ✓ Yes,
    - ✓ No.
    - ✓ I don't know.

11. Monkeypox and chickenpox have similar signs and symptoms?
    - ✓ Yes,
    - ✓ No.
    - ✓ I don't know.

12. Flu-like syndrome is one of the early signs or symptoms of human monkeypox:
    - ✓ Yes,
    - ✓ No.
    - ✓ I don't know.

13. Rashes on the skin are one of the signs or symptoms of human monkeypox:
    - ✓ Yes,
    - ✓ No.
    - ✓ I don't know.

14. Monkeypox is diagnosed by taking a swab sample from the lesion for a polymerase chain reaction
    - ✓ Yes,
    - ✓ No.
    - ✓ I don't know.

15. A person does not exhibit symptoms during the incubation period
    - ✓ Yes,
    - ✓ No.
    - ✓ I don't know.

16. Incubation period of Monkeypox lasts between 3-5 days
    - ✓ Yes,
    - ✓ No.
    - ✓ I don't know.

17. Diarrhea is one of the signs or symptoms of human monkeypox:
    - ✓ Yes,
    - ✓ No.
    - ✓ I don't know.

18. Lymphadenopathy (swollen lymph nodes) is one clinical sign or symptom that could be used to differentiate monkeypox and smallpox cases:
    - ✓ Yes,
    - ✓ No.
    - ✓ I don't know.

19. There is a specific treatment for monkeypox:
    - ✓ Yes,
    - ✓ No.
    - ✓ I don't know.

20. Analgesics and antipyretics can be given to manage local pains and fever caused by monkeypox
    - ✓ Yes,
    - ✓ No.
    - ✓ I don't know.

21. Antivirals are required in the management of human monkepox patients:
    - ✓ Yes,
    - ✓ No.
    - ✓ I don't know.

22. Antibiotics are required in the management of human monkepox patients:
    - ✓ Yes,
    - ✓ No.
    - ✓ I don't know.

23. People who got chickenpox vaccine are immunized against monkeypox:
    - ✓ Yes,
    - ✓ No.
    - ✓ I don't know.

24.There is a specific vaccine for monkeypox:
    - ✓ Yes,
    - ✓ No.
    - ✓ I don't know.

Confident in dealing with Monkeypox cases

1. 1. Are you confident to manage monkeypox cases, if any, based on your current knowledge and skills?
    - ✓ Yes,
    - ✓ No.

2. Are you confident to diagnose monkeypox cases based on your current knowledge and skills?
    - ✓ Yes,
    - ✓ No.

3. Are you confident to diagnose monkeypox cases based on the ability of your current facility to do diagnostic test?
    - ✓ Yes,
    - ✓ No.

Thank you for your contribution to this study.
